# Economic evaluation of a clinical protocol for diagnosing emergency patients with suspected pulmonary embolism

**DOI:** 10.1186/1478-7547-4-12

**Published:** 2006-06-27

**Authors:** Elena V Gospodarevskaya, Stacy K Goergen, Anthony H Harris, Thomas Chan, John F de Campo, Rory Wolfe, Eng T Gan, Michael B Wheeler, John McKay

**Affiliations:** 1 Centre for Health Economics, Monash University, Australia; 2 Department of Diagnostic Imaging, Monash Medical Centre, Australia; 3 Emergency Department, Monash Medical Centre, Australia; 4 Biostatistical Consulting Service, Department of Epidemiology and Preventative Medicine, Monash University, Australia; 5 Department of Haematology, Monash Medical Centre, Australia

## Abstract

**Background:**

The objective of this paper is to estimate the amount of cost-savings to the Australian health care system from implementing an evidence-based clinical protocol for diagnosing emergency patients with suspected pulmonary embolism (PE) at the Emergency department of a Victorian public hospital with 50,000 presentations in 2001–2002.

**Methods:**

A cost-minimisation study used the data collected in a controlled clinical trial of a clinical protocol for diagnosing patients with suspected PE. Thenumber and type of diagnostic tests in a historic cohort of 185 randomly selected patients, who presented to the emergency department with suspectedPE during an eight month period prior to the clinical trial (January 2002 -August 2002) were compared with the number and type of diagnostic tests in745 patients, who presented to the emergency department with suspected PE from November 2002 to August 2003. Current Medicare fees per test were usedas unit costs to calculate the mean aggregated cost of diagnostic investigation per patient in both study groups. A t-test was used to estimate the statistical significance of the difference in the cost of resources used for diagnosing PE in the control and in the intervention group.

**Results:**

The trial demonstrated that diagnosing PE using an evidence-based clinical protocol was as effective as the existing clinical practice. The clinical protocol offers the advantage of reducing the use of diagnostic imaging, resulting in an average cost savings of at least $59.30 per patient.

**Conclusion:**

Extrapolating the observed cost-savings of $59.30 per patient to the wholeof Australia could potentially result in annual savings between $3.1 million to $3.7 million.

## Background

In 1995 the National Health and Medical Research Council announced its commitment to developing evidence-based clinical practice guidelines "to promote best practice linked to outcomes and effective cost management". However, the uptake of evidence-based clinical practices has not been free of problems [1]. Evidence-based medicine is typically promoted on the basis of superior clinical outcomes while the costs of changing to it, borne by individual organizations and practitioners, are downplayed in favour of population benefits [2]. Although there is a growing number of publications emphasising the importance of conducting evaluations of clinical guidelines for diagnosis and prophylaxis of pulmonary embolism and deep vein thrombosis [3], evaluations are either confined to clinical outcomes [4-6] or based on the secondary use of data from the literature rather than on prospective data collection [7,8]. This study is an economic evaluation of a clinical protocol for diagnosing patients with suspected PE based on the data collected in the course of a clinical controlled trial. The purpose of the study is to demonstrate how individual hospitals can achieve an effective cost management through introduction of the best practice for diagnosing emergency patients with suspected pulmonary embolism (PE).

Venous thromboembolic disease, which includes PE and deep venous thrombosis, is the third most common acute cardiovascular disease, exceeded only by cardiac ischemia and stroke. In the United States, the incidence of PE has been estimated to be approximately 1/1000 persons per year [9] and 17 – 26% of untreated patients with PE may die [10,11]. PE – related mortality can be dramatically reduced with anticoagulation therapy [12]. However, because making or excluding the diagnosis of PE based upon clinical evaluation alone is unreliable [13], diagnostic imaging testing of some type is used for this purpose. Diagnostic imaging of the deep veins of the lower limbs or the pulmonary arterial circulation, either with ventilation perfusion (VQ) lung scanning, Doppler ultrasound (US) of the lower limb veins, digital subtraction angiography (DSA) or, more recently, computed tomographic pulmonary angiography (CTPA) have been used to diagnose PE. Unfortunately, the most commonly used tests, VQ and CTPA, have substantial rates of false negative and indeterminate results [14-16]. This leads either to increasingly expensive and invasive investigations that, with the exception of US, also involve ionising radiation or anticoagulation therapythat may be unnecessary.

In recent years, it has been recognized that measurement of the degradationproducts of fibrin (D-dimer) circulating in plasma can be useful in ruling out PE if the levels of D-dimer are low and the pre – test likelihood of PEis low. D-dimer assay is a relatively inexpensive test, which is performed on a small sample of venous blood drawn from the patient. The number of such assays available on the market has rapidly increased, and the sensitivityof the various assays ranges from 80% – 100%. The variability in sensitivity of the assays has probably been responsible for the lack of widespread clinical uptake of D-dimer as a way of excluding PE. When a less sensitive assay yields a negative result in a patient at high risk of VTE, the post test probability of PE may be too high to allow anticoagulation to be withheld [17-19].

However, combination of standardized clinical risk assessment with D-dimer has the potential to be a powerful and non-invasive way of excluding PE which is associated with a high negative predictive value for PE. The two major published strategies for ruling out PE based on the combination of clinical risk assessment and negative D-dimer were developed by Wells et al[18] and Kline et al [19] and more recently Kline and Wells [20].

Wells' strategy has been independently validated [17] and was shown to havea few disadvantages such as classifying a majority of patients as being at intermediate risk of PE, which mandated further investigation with imaging. In addition, the Wells protocol involved a degree of subjective judgement of the likelihood of PE relative to other diagnoses, which is associated with an elevated inter-observer variation in the level of risk assigned to patients.

The strategy suggested by Kline et al [19] had not been prospectively tested until recently [21]. However, its simplicity and relative lack of subjectivity make it attractive for use in an environment, such as the emergency department of a teaching hospital, where the level of clinical experience and training is variable.

In 2002–2003, the Department of Diagnostic Imaging in cooperation with the Emergency and Haematology Departments of Monash Medical Centre, a Victorian720 bed teaching hospital with 50,000 annual emergency presentations, conducted a trial of a clinical protocol for diagnosing patients with suspected PE based on the strategy suggested by Kline et al [19], which is explained below.

**Figure 1:**
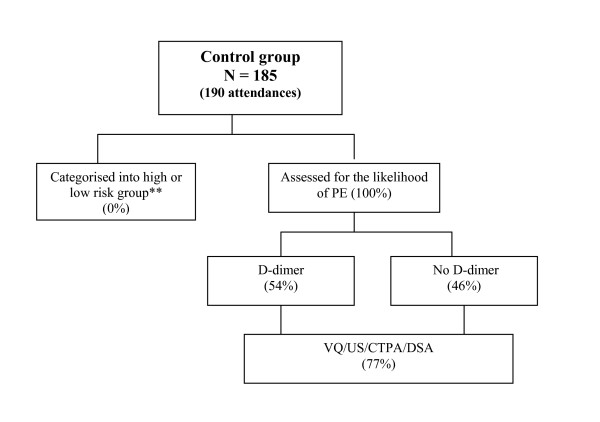
Assessment and diagnosis procedures in the control group* *See the attached file Figure 1 and Figure 2*. *Use of diagnostic tests (%) is calculated with respect to the number of attendances **Categorisation into the low or high risk group (i.e. completing an assessment according to the protocol) is defined as answeringall six questions concerning physiological parameters and medical history that are recorded on the specially designed assessment card, which also contained a request form for imaging or D – dimer, if applicable according to the rules of the structured decision tool [19]. The protocol-specific assessment did not occur for any of the control subjects and thus the risk profile for the control population is unknown.

In the 12 months prior to the clinical trial more than 1,000 patients were assessed for suspected PE in the Emergency Department. The likelihood of PEwas first estimated on the basis of medical history and physical examination and a D-dimer assay was ordered in about 50% of the cases (see Figure 1 below). However, the sensitivity of the test was not seen as high enough towithhold treatment, and subsequent to a negative outcome of D-dimer, an imaging test (typically a VQ) was also requested. The results of VQ can be indeterminate in up to 70% cases [14,22]. Interpreting these diagnostic tests independently of any pre-test clinical assessment added to the high rate of requests for diagnostic imaging and increased the cost of assessment.

Implementation of a protocol for the risk assessment of PE, which combines the standardized clinical risk assessment with D-dimer, has resulted in thereduced use of diagnostic imaging tests without any additional risk of misdiagnosing PE. Clinical risk of the protocol was assessed by calculatingthe negative predictive value of PE in the patients with both "low risk" and a negative D-dimer result.

The objective of this paper is to use the data of the clinical trial to estimate the amount of cost-savings to the Australian health care system ofan evidence-based clinical protocol for diagnosing emergency patients with suspected pulmonary embolism (PE). The detailed description of the original clinical trial can be found elsewhere [21].

## Methods

### Study groups

The intervention group consisted of 745 patients with 789 presentations to the emergency department with suspected PE between November 2002 and August 2003. Historical controls were selected randomly from a group of patients investigated for PE in the emergency department (ED) in the 8 months (January 2002 -August 2002) preceding the study and this group consisted of 185 patients with 190 attendances.

All patients aged 18 or over who attended the ED during the study period and who were thought to have possible PE were potential study subjects. This included patients who had either a D-dimer, VQ, CTPA, or lower limb venous US performed and their symptoms suggested PE rather than deep venous thrombosis alone. This was established on the basis of clinical notes written on the pathology and/or imaging request forms, or if these were notsufficient, study coordinators investigated patients' medical records. The principal investigator (SG) read 5% of medical records for included and excluded patients and there was 92% agreement between the principal investigator and the study coordinators about the outcomes.

Patients who were taking anticoagulant medication on presentation to the EDor who were found to have received it during the follow up period for reasons other than diagnosed PE or DVT were excluded from the final analysis of the negative predictive value of the combination of low risk and negative D-dimer. This is because these patients had a reason other than the effectiveness of the diagnostic strategy for not developing PE during the 3 months follow up. They were not, however, excluded from initial inclusion in the study group. Pregnancy was not an exclusion criterion nor were previous DVT or PE.

The sample size of the intervention group was calculated to estimate the negative predictive value (NPV) of PE in the "low risk" and negative D-dimer group of patients with 5% precision. We assumed an expected NPV of100% based on 99% observed by Kline et al [19]. After allowing for 20% of loss to follow up and for 15% anticoagulation rate during follow up the required sample size was estimated at 135 patients. Recruitment continued until this number was achieved.

For the comparison between control and study subjects with respect to the proportion of patients who received imaging for PE, it was calculated that 184 control subjects would be needed in order to detect, with 90% power ata 5% level of significance, a 15% reduction in the use of diagnostic imaging from a base of 80%.

### Intervention

The patients were assigned into either a "low risk" or a "high risk" PE category according to the outcomes of the standardised clinical assessment, using the protocol designed by Kline et al [19]. The clinical protocol developed by Kline et al (2002) is a decision tool based on a combination of

a) Measurement of physiological parameters (heart rate, blood pressure, arterial oxygen saturation, calf diameter) and medical history (e.g. pre-existing lung disease, haemoptysis, history of general anaesthesia in the preceding month)

b) D-dimer assay result

Compared to other strategies it has clear and simple decision-making rules, a high level of inter-rater reliability (90%) and a 99% NPV for PE (for the combination of low clinical risk and negative D-dimer).

Clinicians working in the Emergency Department were trained to perform riskassessments using a decision tool suggested by Kline et al [19] for all patients whom they suspected may have PE and to document the result (high or low risk) on a form designed for the trial. The form contained the six questions comprising the decision tool and an imaging request to be filled if the conditions of the protocol were met. During the study period, completion of the form was required before imaging would be provided for EDpatients with suspected PE.

Patients categorized as "low risk" were to have a D – dimer assay as the first diagnostic test. The clinicians were instructed not to request pulmonary vascular imaging (VQ, CTPA, lower limb venous ultrasound, or DSA)if the result of the assay for the "low risk" group was negative, as these patients were at extremely low risk of PE. Only those patients from the "low risk" group for whom a D-dimer assay proved positive were to proceedto further diagnostic imaging, starting with a VQ scan. Patients assigned to the "high risk" group did not require a D-dimer assay according to the protocol, but were supposed to receive imaging, with a VQ scan as the firstdiagnostic test.

**Figure 2:**
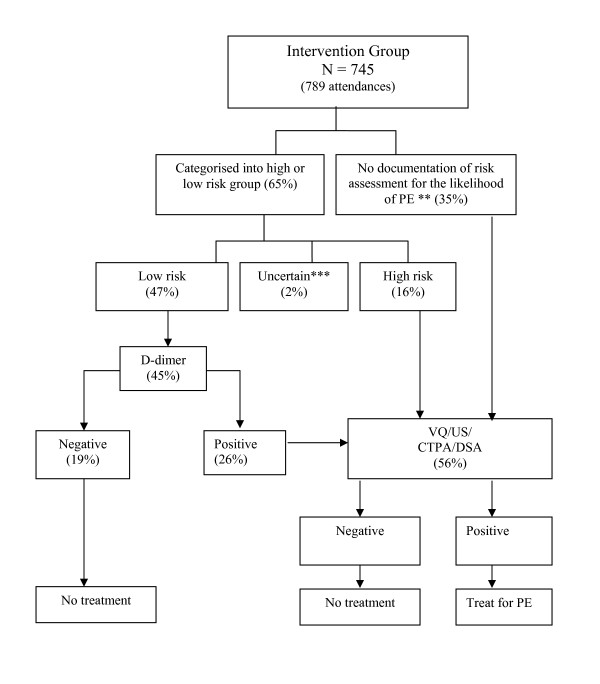
Decision tool for high risk and low risk assessment groups*. *See the attached file Figure 1 and Figure 2*. * Use of diagnostic tests (%) is calculated with respect to the number of attendances **Risk assessment in 35% of the patients presented with suspected PE may or may not have occurred but did not involve completion of risk assessment cards containing the structured decision tool. With respect to these patients it is uncertain whether the use of diagnostic resources has been influenced by the rules outlined in the protocol. ***During the intervention period an assessment involving a structured decision tool was performed on 513 (65%) of attendances although a *valid *assessmentof low or high risk was only attained for 491 (62%) attendances due to the incomplete answers to six questions concerning physiological parameters andmedical history that are recorded on the specially designed assessment card. This resulted in the uncertain outcome of the assessment for 22 (2%) attendances.

Figure 1 and Figure 2 describe the assessment and diagnostic procedures forthe suspected pulmonary embolism in the control and study groups respectively.

Resource use was first estimated in natural units (the number of diagnostictests by test category ordered in the control and intervention groups). Themonetary value of the resources was obtained by using the Medicare Benefit Schedule fees as unit costs (Table 1).

**Table 1:** Diagnostic test unit costs from MBS fee.

**Code**	**Diagnostic Test**	**85% of MBS fee**	**100% of MBS fee**
65120	D-dimer	$11.65	$13.65
61348	Ventilation perfusion (VQ) lung scan	$335.55	$386.45
55244	Doppler Ultrasound (US)	$144.05	$169.45
57350	Computerised tomography (CTPA)	$461.20	$512.10

Time spent by staff in preparing the clinical protocol incorporating the decision tool [19] for assessing patients with suspected PE as well as timespent by clinical staff on attending training sessions adds to the cost of intervention. Information on the number of hours spent in preparation and conducting training sessions as well as information on the number and clinical staff category of the attendees was collected. The average hourly rates by staff category were used to estimate the monetary value of time spent on implementation of the clinical protocol.

### Follow up

All patients who were assessed as being "low risk" and who also had a negative on D-dimer assay result were followed up for the three months following their ED attendance to establish if they had been investigated for or diagnosed with DVT or PE during this period of time. They were also asked whether they had received anticoagulation during follow up. The hospital database was also checked to determine if they had re-presented during the follow up period with PE or DVT. Patients were considered lost to follow up after three mailings of the follow up questionnaire and five phone calls(which occurred during the day and evening both during the week and on weekends) failed to make contact. Patients who were lost to follow up had their names submitted to the Victorian Registry of Births, Deaths, and Marriages to determine if they had died in Victoria during the follow up period. It was decided prior to commencement of the study that any deaths which occurred during the follow up period would be assumed to be due to pulmonary embolism unless this possibility could be excluded by post mortem examination.

### Clinical outcome

The combination of low clinical risk and negative D-dimer result wasobserved in 150 attendances by 148 study subjects. Of the 150 attendances, 130 attendances had follow up completed successfully, resulting in a loss to follow up of 13%. Of the 130 subjects successfully followed, 16 received an anticoagulant medication either at the time of their presentation to the ED with suspected PE or at some time during the follow up period. The reason for receiving anticoagulation medication were either previously (prior to the ED presentation) diagnosed thromboembolic disease (e.g. pulmonary embolism) or pulmonary or cerebral thromboembolic complications in patientswho were predisposed to them due to severe cardiac failure or coagulation disorders. No patient with low risk and negative D dimer was commenced on anticoagulants during the follow up period due to diagnosed PE or DVT. Thus, 114 patients satisfied all inclusion and exclusion criteria for evaluation of the accuracy of the combination of the clinical risk assessment and negative D – dimer.

Of these 114 patients with negative D dimer and no anticoagulation, 1 patient (1%) had PE. He was a 43 year old male with a history of cardiac arrhythmias and intermittent medical treatment for the arrhythmias. He had a VQ performed based on the clinical judgement of the attending ED physician, despite being classified as low risk for PE and having a negative D – dimer result and the scan was interpreted as high probability for PE.

Thus the negative predictive value (NPV) of the combination of low risk andnegative D – dimer in our study group was 99% (95% CI = 96% – 100%).

None of the 20 patients lost to follow up were investigated or diagnosed with PE at the hospital (or at the geographically closest public hospital, which was also part of the hospital network) during the 3 month follow up period. An audit of the Victorian Registry of Births, Deaths, and Marriagesrevealed that none of these patients were recorded as having died in the State of Victoria during the follow-up period.

This result confirmed the existing clinical evidence that the risk management strategy of the protocol adopted for the trial excludes almost all cases of clinically significant PE without the need for pulmonary vascular imaging [19,23]. The trial protocol did not introduce any additional clinical risk in comparison with existing practices for diagnosing PE, while it had the advantage of reduced utilisation of expensive diagnostic imaging resources.

### Economic evaluation

The study hypothesis was that upon introduction of an evidence-based protocol there would be no difference in the number of correctly diagnosed PE patients, but that there would be a saving in the use of resources primarily from a reduction in the number of pulmonary vascular imaging tests for patients categorised into a "low risk" group who also had negative D-dimer at the pre-test assessment. In addition, it was expected that there might be a small savings from patients in the "high risk" group by avoiding a D-dimer test.

The objective of the economic evaluation was to estimate the cost-savings resulting from implementation of the evidence-based clinical protocol by comparing the average monetary value of resources used for diagnosis of suspected PE in the control group with the corresponding monetary value of resources used in the intervention group. Economic evaluation was conducted from the perspective of the Australian health care system.

### Cost of resource use

Resource utilisation by category of diagnostic tests for both groups was collected during the clinical trial. Current Medicare Benefit Schedule (MBS) fees for a D-Dimer assay, a VQ scan, a US and a CTPA, were used as unit costs [24]. The Department of Diagnostic Imaging routinely performs these tests for the outpatient group and has advised that the MBS reimbursement rates (85% of schedule fees) adequately reflect the hospital's expenditures. This is probably a conservative estimate of unit costs, so wehave conducted our analysis using both 85% and 100% of MBS fees. These are presented in Table 1. All resource use was aggregated by the category of diagnostic test and valued at current MBS fee per test to create total costs of diagnostic investigation for the control and the intervention groups.

The difference in the total cost of diagnostic tests per patient in the control and intervention groups produced an estimate of the amount of cost-savings due to implementation of the evidence-based protocol.

## Results

Table 2 shows demographic characteristics of the patients in the control and intervention groups.

**Table 2:** Demographic characteristics of patients in the control and intervention groups.

	Control n = 185	Intervention n = 745
Age	56.2 (SD 18.4)	55.0 (SD 19.1)
Gender (% male)	40.5	42.9
Positive diagnosis of PE on imaging* (%)	12.0	9.5

SD = standard deviation

*positive diagnosis of PE on imaging is derived from high probability VQ or positive CTPA or positive lower limb venous ultrasound.

No statistically significant differences were found between the control andintervention groups with respect to mean age (p = 0.30), gender (Pearson Chi – square = 0.33; df = 1; p = 0.62) or positive diagnosis of PE on imaging (Chi-square = 1.1, df = 1; p = 0.29) as a proxy for the prevalence of PE in the study groups. True prevalence of PE in the two groups is unknown as, according to the trial protocol, only the "low risk" patients who were D-dimer negative were followed up to determine if they developed PE during the 3 month period after their initial investigation.

During the trial, risk assessment as outlined in the protocol was performedand documented for 513 (65%) of the attendances, if judged by the number of completed risk assessment cards that were returned to the study coordinators. However, there was anecdotal evidence that the adherence to the protocol was in fact higher, but during periods of high demand the emergency department medical staff did not complete risk assessment cards.

Outcome of the clinical trial confirmed that the evidence-based protocol for diagnosing PE is just as effective (in terms of NPV) as the clinical practice it has replaced. In such instances where the alternative clinical practice is not associated with improved outcomes but is suggestive of reduced utilisation of limited health care resources, such as diagnostic imaging, a cost-minimisation analysis is employed.

Table 3 shows the difference in proportion of diagnostic tests performed inthe control and intervention groups.

**Table 3:** Difference in the proportion of diagnostic tests performed in the control and intervention groups.

	Control n = 185	Intervention n = 745	Absolute risk difference	Pearson Chi-Square Statistics (df = 1)	P-value
D-dimer	54.1%	93.2%	48.9%	181.5	<0.0001
VQ	70.3%	52.9%	-17.4%	18.2	<0.0001
LLUS	20.5%	17.6%	-2.9%	0.87	0.4
CTPA	9.2%	8.1%	-1.1%	0.25	0.6

Digital subtraction angiography was ordered only once in the control group and was excluded from the calculations since this invasive test, which was occasionally used to confirm or exclude PE in patients with high risk and otherwise indeterminate imaging results, has been practically replaced by anon-invasive CT pulmonary angiography (CTPA).

After introducing an evidence-based protocol, the proportion of patients who received diagnostic imaging tests reduced across all categories of tests, although the difference was statistically significant only with respect to VQ scans. As expected, utilisation of D-dimer increased significantly in the intervention group. Table 3 shows that 93.2% of the intervention group patients had a D-dimer test. The majority of patients from the interventiongroup were assigned a D-dimer assay regardless of the outcome of the pre-test assessment, and the expected reduction in the number of D-dimer assays in the "high risk" group was not observed. Although doctors continued performing D-dimer on some patients from the "high risk" group, the larger proportion (75%) of D-dimer assays were performed, appropriately, on "low risk" patients.

Implementation of the evidence-based protocol in the emergency department involved 10–12 hours of preparing educational material and 8–10 one hour small group teaching sessions which involved 15 medical and nursing staff from the emergency department, and eight staff from the Diagnostic Imaging Department, including medical imaging technologists, radiologists and radiology residents at all levels of clinical experience. These sessions consisted of a presentation of the theory surrounding the principle of exclusion of PE using D-dimer, discussion of the problems with imaging tests performed on patients with PE (in particular, indeterminate results) and a detailed discussion of what was required to perform the risk assessment.

Assuming that the hourly rate of the members of the protocol implementation team is $60, the cost of preparing educational material and conducting training sessions is about $1,200. The opportunity cost for the medical and diagnostic imaging staff to attend the sessions is estimated at about $1,800 assuming that the average hourly rate for 23 staff members who attended the training sessions is $80.

Table 4 shows the average costs per patient in the control and interventiongroups using 85% of MBS fees as a lower estimate.

**Table 4:** Average costs of diagnostic tests in the control and intervention groups using 85% of MBS fees ($/per patient).

	Control n = 185 (95%CI)	Intervention n = 745 (95%CI)	Mean cost reduction
**Clinical costs**			

D-dimer	6.30 (5.50–7.10)	10.90 (10.60–11.10)	-4.60
VQ	235.80 (213.50–258.10)	177.50 (165.40–189.50)	58.30
LLUS	29.60 (21.10–38.10)	25.30 (21.40–29.30)	4.30
CTPA	42.40 (23.00–61.80)	37.10 (28.10–46.20)	5.30

**Protocol implementation cost**			

Preparing protocol materials and conducting training sessions	0	1.60	-1.60
Attending training sessions	0	2.40	-2.40
**Total**	314.10 (283.00–345.10)	246.80 (228.60–265.00)	59.30

Applying 85% of MBS fees as unit costs (Table 1), the estimated additionalcost of protocol implementation associated with an increased use of D-dimerassays is $4.60 per patient. However, this cost increase was more than offset by the decreased costs of diagnostic imaging in the intervention group, with the largest reduction in the cost of VQ lung scan of $58.30 per patient. After adjusting for the one-off costs of implementation the protocol, the total cost of diagnostic investigation per patient from the intervention group was $59.30 less than the cost per patient from the control group. The difference was statistically significant (t = 3.1; df = 928; p = 0.002). Applying 100% of MBS fees as unit costs (Table 1), the estimatedreduction in the total cost of diagnostic investigation per patient was $68.70.

The number of presentations with suspected PE to the emergency department of the study hospital is estimated at 1000 patients per year. This suggests an annual cost savings of $59,300 using 85% of MBS fees ($68,700 if 100% of MBS fees are used).

## Discussion

The study found significant cost-savings to the health care system from a reduction in the number of pulmonary vascular imaging tests for patients categorised into a "low risk" group at the pre-test assessment. At the same time the number of D-dimer tests was greater in the intervention groupthan in the control group. Much of this increase was appropriate and in line with the implementation of the evidence-based protocol, as the majority of D-dimer assays were performed on the "low risk" patients. This additional cost has been more than offset by the reduction in the number and cost of diagnostic imaging investigations. Special emphasis during the training sessions regarding the inappropriateness of D-dimer testing in "high risk" individuals may bring additional cost-savings in this area. However, timepressure and financial penalties tied to length – of – stay in the emergency department meant that D dimer was often performed before the doctor managing the patient had time to undertake the risk assessment as the D – dimer result was not available for 1–2 hours after the blood speciment was drawn. This represents an organizational barrier to performing the risk assessmentstrategy prior to diagnostic testing.

It can be argued that the cost savings are associated with an increased time of risk assessment in the intervention group. However, although no formalcategorisation of the control subjects into "high" and "low" risk grouptook place prior to the protocol implementation, the informal risk assessment of the control subjects with suspected PE was carried out by theED staff. This involved the use of the ED resources in terms of clinicians'time spent on obtaining medical history, conducting physical examination, writing pathology request, interpreting the test outcome etc. The decision rule introduced in the protocol is associated with one essential difference- that ordering and interpreting of the outcome of a D-dimer diagnostic test is no longer occurs independently of any structured pre-test clinical assessment.

Therefore it can be argued that no significant opportunity cost was lost as a result of protocol implementation, however there is a possibility of a small marginal cost associated with time spent on recording the answers to 6 questions of the structured decision tool on the specially designed. This extra time of about 2–5 minutes is believed to be too small to significantly change the outcome of the study. In addition, it is also possible, although there is no way of proving this on the basis of collected data, that the decision rule may have reduced the time involved in patient assessment by focussing the emergency medical staff on a few clinical findings which were the most important in determining pre-test risk.

The use of a non-random historic control group is a limitation of the study. However it was not feasible to have within a single department two concurrent groups of medical practitioners randomised to different training programs or to assign patients to staff with different training. Nevertheless, there was not a significant gap in time between the study groups and they appear to be comparable in terms of risk of PE. The exact risk profile of PE in the control group is unknown, as the risk assessment according to the decision tool was not performed on them. It is probably reasonable to assume, however, that it was similar to that of the intervention group given the similar demographic profiles of the two groups.

The implemented risk assessment strategy has gained a high degree of acceptance by the ED medical staff of the trial hospital and is now used as the first line "test" to determine the requirement for imaging. This has reduced the demand for imaging of patients with suspected PE to a significant degree. More specific D-dimer assays, which may become available in future, would allow even further reduction in the need for imaging as there would be fewer patients with false positive D-dimer results who required imaging to rule out PE.

The study site is reasonably representative of large emergency departments in Australia and there is no reason to believe that these results could not be replicated in other sites. If they were, there is the potential for substantial cost savings with no increase in risk. In the absence of national or state-wide data on the number of patients presenting to an ED with PE we have estimated the annual national savings on the assumption that the trial hospital is representative of public hospitals with an ED. With 50,000 presentations in the trial hospital [25] the annual total cost savings for 1000 patients with suspected PE was about $60,000. Total Victorian presentations to ED were 705,000 [25]. The projected savings for Victoria are calculated as 705,000/50,000 × $60,000. The Victorian public hospital system represents some 27% of public hospital separations in Australia, suggesting a potential national annual cost savings from the introduction of a clinical protocol for diagnosing emergency patients with suspected PE of between $3.1 million and $3.7 million depending on which unit cost of the diagnostic tests is used.

## Conclusion

It is possible to implement a combination of bedside clinical risk assessment and D-dimer assay in low risk emergency department patients to exclude pulmonary embolism. The result is a reduction in the use of imaging with significant cost savings with no increase in health risks for these patients.

## Competing interests

This work was supported by a Targeted Grant from the National Institute of Clinical Studies (NICS), the Australian organization for bridging the gap between evidence and practice.

The authors have no relationship with the NICS apart from being the recipients of a research grant which allowed this project to be undertaken. The NICS neither controlled nor influenced the study design, data collection, analysis, interpretation, writing or submitting of the article.

All authors declare that they are unaware of any real or potential conflicts of interests arising from their involvement in the research project described in this manuscript or in their roles as co-authors of the manuscript.

## References

[B1] Hirst GH, Ward JE (2001). Clinical practice guidelines: reality bites. MJA.

[B2] Coiera E (2001). Maximising the uptake of evidence into clinical practice: an information economics approach. MJA.

[B3] Wittkowsky AK (2004). Effective anticoagulation therapy: defining the gap between clinical studies and clinical practice. American Journal of Managed Care.

[B4] Henschke CI, Yankelevitz DF, Mateescu I, Whalen JP (1994). Evaluation of competing tests for the diagnosis of pulmonary embolism and deep vein thrombosis, Part I. Clinical Imaging.

[B5] McEleny P, Bowie P, Robins JB, Brown RC (1998). Getting a validated guideline into local practice: implementation and audit of the SIGN guideline on the prevention of deep vein thrombosis in a district general hospital. Scott Med J.

[B6] Durieux P, Dhote R, Meyniard O, Spaulding C, Luchon L, Toulon P (2001). D-dimer testing as the initial test for suspected pulmonary embolism. Appropriateness of prescription and physician compliance to guidelines. Thromb Res.

[B7] Wade WE, Chisholm MA (2000). Venous thrombosis after acute spinal cord injury: cost analysis of prophylaxis guidelines. American Journal of Physical Medicine & Rehabilitation.

[B8] Brick RL (2000). Proficient and cost-effective approaches for the prevention and treatment of venous thrombosis and thromboembolism. Drugs.

[B9] Siverstein MD, Heit JA, Mohr DN, Petterson TM, O'Fallon WM, Melton LJ (1998). Trends in the incidence of deep vein thrombosis and pulmonary embolism: a 25 year population – based study. Arch Intern Med.

[B10] Goldhaber SZ, Visani L, De Rosa M (1999). Acute pulmonary embolism: clinical outcomes in the International Cooperative Pulmonary Embolism Registry (ICOPER). Lancet.

[B11] Barrit DW, Jordan SC (1960). Anticoagulant drugs in the treatment of pulmonary embolism. Lancet.

[B12] Douketis JD, Kearon C, Bates S, Duku EK, Ginsburg JS (1998). Risk of fatal pulmonary embolism in patients with treated venous thromboembolism. JAMA.

[B13] Kearon C, Ginsberg JS, Hirsh J (1998). The role of venous ultrasonography in the diagnosis of suspected deep venous thrombosis and pulmonary embolism. Ann Int Med.

[B14] Robinson PJ (1996). Ventilation-perfusion lung scanning and spiral computed tomography of the lungs: competing or complementary modalities?. Eur J Nucl Med.

[B15] Safriel Y, Zinn H (2002). CT pulmonary angiography in the detection ofpulmonary emboli: a meta-analysis of sensitivities and specificities. Clin Imaging.

[B16] Musset D, Parent F, Meyer G, Maitre S, Girard P, Leroyer C, Revel MP, Carette MF, Laurent M, Charbonnier B, Laurent F, Mal H, Nonent M, Lancar R, Grenier P, Simonneau G (2002). Diagnostic strategy for patients with suspected pulmonary embolism: a prospective multicentre outcome study. Lancet.

[B17] Perrier A, Bounameaux H, Morabia A, De Moerloose P, Slosman D, Didier D, Unger PF, Junod A (1995). Diagnosis of pulmonary embolism by decision analysis-based strategy including clinical probability, D-Dimer levels and ultrasonography: a management study. Archives of Internal Medicine.

[B18] Wells PS, Anderson DR, Rodger M, Stiell I, Dreyer JF, Barnes D (2001). Excluding pulmonary embolism at the bedside without diagnostic imaging: management of patients with suspected pulmonary embolism presenting to the emergency department by using a simple clinical model and d-dimer. Ann Intern Med.

[B19] Kline JA, Nelson RD, Jackson RE, Courtney MD (2002). Criteria for the safe use of D-dimer testing in emergency department patients with suspectedpulmonary embolism: A multicenter US study. Ann Emerg Med.

[B20] Kline JA, Wells PS (2003). Methodology for a rapid protocol to rule out pulmonary embolism in the emergency department. Ann Emerg Med.

[B21] Goergen SK, Chan T, deCampo JF, Wolfe R, Gan E, Wheeler M, McKay J (2005). Reducing the use of diagnostic imaging in patients with suspected pulmonary embolism: validation of a risk assessment strategy. Emergency Medicine Australasia.

[B22] Hull RD, Raskob GE, Coates G, Panju AA, Gill GL (1989). A new non-invasive management strategy for patients with suspected pulmonary embolism. Archives of Internal Medicine.

[B23] Kline JA, Israel EG, Michelson EA, O'Neil BJ, Plewa MC, Portelli DC (2001). Diagnostic accuracy of a bedside D-dimer assay and alveolar dead-space measurement for rapid exclusion of pulmonary embolism: a multicenter study. JAMA.

[B24] The Medicare Benefit Schedule. http://www7.health.gov.au/pubs/mbs/index.htm.

[B25] State Government of Victoria, Department of Human Services, Emergency Demand Coordination Group (2002). Hospital admission risk program (HARP) Background paper Melbourne.

